# Otomycosis in Central Iran: A Clinical and Mycological Study

**Published:** 2011-12-01

**Authors:** B Barati, S A R Okhovvat, A Goljanian, M R Omrani

**Affiliations:** 1 Department of Otolaryngology, Shahid Beheshti University of Medical Sciences, Tehran, Iran; 2 Department of Otolaryngology, Isfahan University of Medical Sciences, Isfahan, Iran

**Keywords:** Otomycosis, Aspergillus, Candida, Pruritus, Iran

## Abstract

**Background:**

Otomycosis is a fungal infection of the external ear with bothersome symptoms. The aim of this study was to evaluate the prevalence of fungal agents, predisposing factors and characteristics of patients.

**Methods:**

Between May 2008 and April 2010, 171 patients with clinical suspicion of otomycosis were enrolled and the samples from their external ear were examined for any mycological infection.

**Results:**

Otomycosis was confirmed after mycological diagnosis in 69% of clinically suspected patients. The highest incidence of otomycosis was in autumn and in patients aged 21-40 years old. Working in dry dusty environment was a major predisposing factor. Pruritus was the most common symptom. Aspergillus flavus was the most common fungus in otomycosis followed by A. niger, Candida albicans, A. fumigatus, A. nidulans and C. parapsilosis.

**Conclusion:**

Clinical suspicion of otomycosis is important to prevent unnecessary use of antibiotics. Etiology of fungal pathogens in dry dusty regions is not similar to hot humid areas and this needs to be considered in future susceptibility tests and treatment of patients with otomycosis.

## Introduction

Otomycosis also known as fungal otitis externa is a fungal infection often involving the pinna and the external auditory meatus, however in the presence of a perforated tympanic membrane, it can also involve the middle ear.[1][2][3][4] The mastoid cavity can also be involved following open cavity mastoidectomy.[5] The main symptoms include pruritus, otalgia, aural fullness, hearing impairment, otorrhea and tinnitus.[6][7][8][9]

The prevalence of otomycosis is related to the geographic area with higher rates in tropical and subtropical climates.[4] Predisposing factors include alterations in immunity, use of steroids, dermatological diseases, loss of cerumen, use of broad-spectrum antibiotics and hearing aids.[3][9][10][11][12]

Literature search reveals that most of studies about the etiology of otomycosis have been carried out in tropical and subtropical areas. Our study was carried out to evaluate clinical and mycological features of otomycosis in a dry area of Iran.

## Materials and Methods

The samples were obtained from 171 consecutive patients attending the Otolaryngology Clinic of Isfahan University of Medical Sciences, between May 2008 and April 2010 with the clinical diagnosis of otomycosis. The clinical diagnosis was made based on pruritus, otalgia, and aural fullness, hearing impairment, otorrhea and tinnitus.

The samples were obtained using a microscope and sterile cotton swabs and were sent to the Mycology Department, Isfahan University of Medical Sciences, using sterile means of transport. All samples were initially conserved between 2 and 8°C to aviod overgrowth of commensal saprophytic flora. Refrigeration was carried out in 4 samples .This was carried out when technical problems prevented an immediate transfer of samples to the laboratory or mycological examination could not be performed immediately in the laboratory .When refrigeration was necessary, it was never used for more than 48 hours.

The samples were sown in the laboratory and inoculated on three different media: Sabouraud chloramphenicol agar, blood agar and Malt yeast agar. Each sample swab was rolled on all three media and inoculated in a heater at 30°C for 2 weeks. Using the Loddler´s[13] and Hoog and Guarro criteria,[14] mycological identification was made.

## Results

One hundred and seventy one patients with the clinical diagnosis of otomycosis were evaluated; among them, 86 patients (50.3%) were females and 85 (49.7%) were males. The average age of patients was 35.8 (9-78) years old. Patients in their fourth decade of life made up the biggest group (30.4%) followed by 21-30 age group (22.2%).

Construction workers and farmers (working in dry dusty environments) made up the biggest group (61.1%) while among male and female patients, housewives and farmers were the biggest group (73.2%). The seasonal distribution was 36.8% in autumn, 30.4% in summer, 18.1% in winter and 14.6% in spring. Male to female ratio was highest in autumn (54% versus 46%) and lowest in summer (40% versus 60%).

Of 171 patients, no organism was isolated in 10 patients (5.8%) and bacterial pathogens were isolated in 43 (25.1%) subjects. In 118 patients (69%), mycological isolation was positive. The most common fungal isolates belonged to the species of Aspergillus accounting for 91.5% of all fungal isolates. Out of 108 Aspergillus positive samples, A.flavus was the most common (49%), followed by A.niger (41.6%), A.fumigatus (5.5%) and A.nidulans (3.7%). Species of Candida constituted 8.5% of fungal isolates. Other fungal species like Penicillium were not isolated (Table 1). Staphylococcus species were reported as the dominant microbial pathogen in 24 patients (14%), Pseudomonas aeruginosa in 9 patients (5.3%) and in 10 patients (5.8%) mixed flora was reported.

Six patients (5%) were regular swimmers and previous use of ototopical antibiotics/steroids was revealed in 25 patients (14.6%). None of our patients was immunocompromised (receiving chemotherapeutic agents or systemic steroids). In the current study, we did not encounter silent perforations of the tympanic membrane complicated by fungal infections of the external ear canal. All female patients mentioned wearing head covering regularly. Pruritus (65%) was the most common symptom followed by otalgia (55%), ear fullness (46%), otorrhea (40%) and hearing loss (33%).

**Table 1:** Results of mycological examination in clinically suspected otomycosis.

**Fungal species**	**Otomycosis No. (%)**	**Gender (F:M)**
*A. flavus*	53 (49)	25:28
*A. niger*	45 (41.6)	25:20
*C. albicans*	9 (7.6)	6:3
*A. fumigatus*	6 (5.5)	3:3
*A. nidulans*	4 (3.7)	1:3
*C. parapsilosis*	1 (0.9)	1:0
Total	118	61:57

## Discussion

Otomycoses is frequent in tropical and subtropical climates because of heat and humidity.[11][15][16] Diagnosis of otomycosis is usually made by clinical findings with pruritus being the most common symptom followed by otalgia.[3][6][8] In this study, presumed diagnosis of otomycosis was confirmed by laboratory findings in 69%. Aneja et al.,[17] reported 78% of the patients positive for otomycosis, Kaur et al.,[18] reported otomycosis in 74.7% patients, Ozcan et al.,[18] in 65% patients and Chin and Jegathesan[19] in 74.6% patients. Pontes et al.,[6] reported otomycosis in 19.4% patients.

Higher incidence of otomycosis was reported in females than males in previous studies [6][17][18][20] similar to the findings of our stydy. Highest prevalence of otomycosis in summer has been reported by Paulose et al.,[5] Ozcan et al.,[18] Ghiacei et al.,[20] and Pontes et al.[6] However, seasonal distribution in our study was highest in autumn probably due to dry dusty winds in this season. Male to female ratio was also highest in autumn and those working in dusty environments (construction workers and farmers) were the biggest group among male patients.

In this study, the species of Aspergillus were the largest taxon isolated from patients. A. flavus was the most common fungal pathogen followed by A. niger, A. fumigatus and A. nidulans. Araiza et al.,[21] also reported A. flavus to be the most common pathogen in Mexico City. This was different from studies conducted in hot humid regions where A. niger was the most common mycological pathogen.[11][17][18][22][23] Kaur et al.,[8] reported A. fumigatus as the most common cause of otomycosis. Darko et al.,[24] and Pontes et al.,[6] reported Candida genus as the predominant pathogen in otomycosis.Occurence of A. nidulans in our region with dry dusty winds was not reported in hot humid areas.[17][18][19] Aspergillus species are common saprophytic organisms in the environment. The human external auditory canal is an ideal environment for this fungus to grow and abundance of proteins, carbohydrates, favorable humidity and temperature explain this finding. [25] Other fungal species like Penicillium were not isolated in our study.

Otomycosis was seen more frequently between the age group 21-40 years old and had a higher incident in females than males, a finding similar to that of Aneja et al.,[17] Fasunla et al.,[9] and Pontes et al.,[6] Earlier studies from hot humid areas had considered wearing head covering as a predisposing factor in otomycosis. [5][15][17][18] In the current study, all female patients regularly wore head covering and we could not confirm it as a possible predisposing factor .Swimming was revealed in 5% of patients while others have reported higher rates and considered it as a predisposing factor for otomycosis.[5][9][17][18][24]

Clinical suspicion of otomycosis can prevent unnecessary use of antibiotics and mycological confirmation of otomycosis in 69% of patients indicating the importance of proper clinical diagnosis. Etiology of fungal pathogens in dry dusty regions is not similar to hot humid areas and this needs to be taken into account in the treatment of patients. Further research is needed to determine the susceptibility of fungal agents and appropriate treatments.
